# Laboratory Investigation of Indigenous Consortia TERIJ-188 for Incremental Oil Recovery

**DOI:** 10.3389/fmicb.2018.02357

**Published:** 2018-10-09

**Authors:** Neha Sharma, Meeta Lavania, Vipin Kukreti, Dolly Pal Rana, Banwari Lal

**Affiliations:** ^1^Microbial Biotechnology, Environmental and Industrial Biotechnology Division, The Energy and Resources Institute, New Delhi, India; ^2^Institute of Reservoir Studies, Oil and Natural Gas Corporation Limited, Ahmadabad, India

**Keywords:** microbial enhanced oil recovery, indigenous consortia, Thermoanaerobacter sp., biomass, core flood assay

## Abstract

Bacterial Profile modification is an efficient process which brings the alteration in permeability of the porous media of the reservoir by selective plugging which eventually recover the residual oil. It is an advantageous and feasible method for residual oil recovery from high permeability zones of the reservoir. In this study, indigenous bacterial consortia, TERIJ-188 was developed from Gujarat oil fields. TERIJ-188 was identified as *Thermoanaerobacter* sp., *Thermoanaerobacter brockii, Thermoanaerobacter italicus, Thermoanaerobacter mathranii, Thermoanaerobacter thermocopriae.* The novelty of consortia was that it produces biomass (850 mg l^-1^), bio-surfactant (500 mg l^-1^), and volatile fatty acids (495 mg l^-1^) at 70°C in the span of 10 days, which are adequate to alter the permeability and sweep efficiency of high permeability zones facilitating the displacement of oil. The biosurfactant was analyzed for its functional group by FTIR and NMR techniques which indicate the presence of C-N bond, aldehydes, triacylglycerols. TERIJ-188 showed an effective reduction in permeability at residual oil saturation from 28.3 to 11.3 mD and 19.2% incremental oil recovery in a core flood assay. Pathogenicity test suggested that TERIJ-188 is non-toxic, non-virulent and safe for field implementation.

## Introduction

Recent oil recovery technologies depend on natural reservoir pressure and/or injection of gas or water into the reservoir to drive the oil to producing wells (Van Hamme et al., 2003; Belyaev et al., 2004). However, these technologies mobilize only about 30–50% of the residual oil contained in the reservoir (Sabatini et al., 2000; Al-Wahaibi et al., 2014). Microbial enhanced oil recovery is potentially low-priced technique in which different microorganisms and their metabolic products are convinced to exploit the remaining trapped oil in the reservoir.

Lack of sufficient natural drives in most of the reservoir in the world has to lead the practice of supplementing the natural reservoir energy by some form of artificial drive, the most basic method being the injection of displacement fluid like water or gas. The problem associated with these recovery mechanisms is channeling of the displacement fluid through high permeable streaks thus resulting in a poor sweep efficiency of the oil. In such cases, bacterial profile modification is being carried out to reduce high permeability streaks using conventional chemical-based techniques such as gels and polymers. Microbes provide another environment-friendly noble technique from the field of biotechnology to improve the sweeping efficiency of oil from the water flooding reservoir. Since the water flood is naturally sought the zone of least resistance (or highest permeability), low permeability zones are bypassed. After sometime, recoverable oil is “watered out” of the high permeability zones, but the low permeability streaks still contain sizable residual oil. Therefore “Bacterial Profile modification” is the most effective practice which provides to recover the residual oil from lower permeability zones.

**FIGURE 1:**
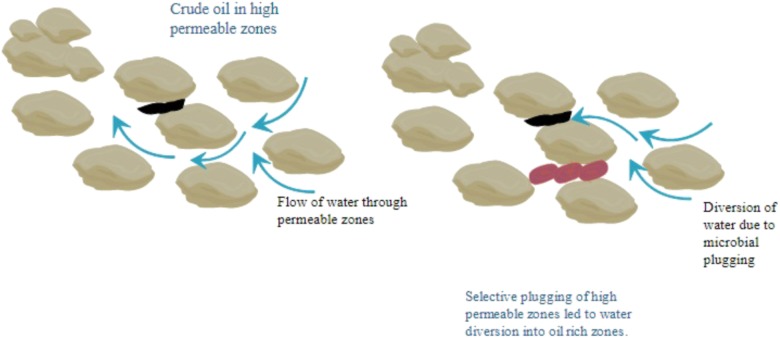
Schematic of bacterial profile modification.

Microbial Enhanced Oil Recovery (MEOR) is widely applicable in sandstone (Bryant et al., 1994) and carbonate reservoirs (Zekri et al., 2003) with light/heavy crude oil (Feng et al., 2005; Dawei et al., 2012) and low/mid and high permeability (Guo et al., 2007; Long et al., 2013). Numerous satisfactory results of field trials lead to the fact to contemplate MEOR as an adequate alternative for other Improved Oil Recovery/Enhance Oil Recovery technologies. Bacterial Profile Modification (BPM) is a process where selective microbes along with nutrients are preferentially entered in the reservoir along with high permeability thief zones. Microbes grow and produce biopolymers which plug the high permeability zones. Permeability reduction results in the diversion of displacement fluid toward low permeability zone, which provide better sweep efficiency of oil lead to enhanced oil recovery (Guo et al., 2015). *In situ* microbial injection also creates a biomass which reduces the pore throat size and in turn reduces the permeability of watered out thief zones or high-permeability streaks. Permeability reduction results in the diversion of displacement fluid toward low permeability zone, which provide better sweep efficiency of oil (**Figure 1**).

MEOR has various advantages over other chemical or mechanical processes which involve cost-effectiveness, limited utilization of thermal energy and less toxicity. Moreover, it does not rely on the price of crude oil (Fox and Bala, 2000; McInerney et al., 2002; Maneerat, 2005; Nitschke et al., 2005; Mukherjee et al., 2006; Nitschke and Pastore, 2010; Lima et al., 2011a,b; Ghojavand et al., 2012; Gudina et al., 2013; Xia et al., 2013). The present study is to investigate Enhanced Oil Recovery by BPM where the objective is to reduce high permeability zones of the oil reservoir by selective plugging. To target this, a range of measures was taken; development and characterization of indigenous bacterial consortia for optimum metabolites production [biomass, bio-surfactant, and volatile fatty acids (VFA)]. Potential consortia TERIJ-188 was further evaluated in core flood studies for enhanced oil recovery in which it was injected into the core and then a gradual reduction in the absolute permeability of the core indicating selective plugging of permeable zones within the core. The microbial processes “elucidated by consortium TERIJ-188 both in terms of targeted residual oil recovery and by-product formation, establish its value as an economical and environmentally attractive option.”

## Materials and Methods

### Site Description

The oil and formation fluid were collected from oil wells located in Ahmedabad (Gujarat), India, at a longitude 23.22°N 72.68°E with the elevation of 81 m (266 ft). The average annual rainfall is around 803.4 mm (31.63 inches). The climate is dry and hot with the maximum 42°C and minimum 14°C temperature. The *in situ* bottom hole temperature of the reservoirs were in the range of 68–75°C.

### Sample Collection

Crude oil and formation fluid samples were collected from the wellhead of different oil wells (Kalol, K #35; K#655 and Jotana, J#188, J#31, J#06) after connecting lines were flushed for 5 mins prior to filling a pre-sterilized serum bottles containing 1 ml of 2% Na_2_S during July, 2015 as described by Lavania et al. (2015). Formation fluids were transported at ambient temperature to the laboratory within 24–48 h, stored at 4°C and processed immediately for physico-chemical measurements and microbial analysis.

### Physicochemical Characterization of Formation Fluid/Oil

Physico-chemical properties were measured as described by Lavania et al. (2015). Heavy metal content of the formation fluids included arsenic (As), cadmium (Cd), chromium (Cr), copper (Cu), zinc (Zn), silver (Ag), nickel (Ni), and Iron (Fe) with the detection limits of 0.01, 0.004, 0.01, 0.02, 0.05, 0.05, and 0.03 mg l^-1^, respectively. The presence of anions (chloride, fluorides, and sulfate) was also determined. The carbon, hydrogen, nitrogen, and sulfur (CHNS) composition of formation fluids was determined using IS: 1350/American Public Health Association guideline (**Table 2**). The amount of total petroleum hydrocarbons (TPH) recovered from oil was determined gravimetrically as described by Lavania et al. (2015). All the solvents, reagents used in the study were of analytical grade and obtained from commercial suppliers.

### Enrichment of Thermophilic Consortia

To isolate thermophilic anaerobic microbes for BPMs, 10% (v/v) of formation fluid were inoculated into pre-sterilized serum bottles containing a modified Baltch medium. The modified Baltch medium per liter contains NH_4_Cl, 2g: MgSO_4_.7H_2_O, 0.2; NaHCO_3_, 2.6g; Molasses, 10g; NaCl, 1g; and L-cysteine, 0.5 g. The pH of the media was adjusted to 8.0. In media preparation a volume of 100 μl of resazurin (oxygen indicator) was added in 1000 ml medium and boiled under a nitrogen purge conditions to remove dissolved oxygen (till media turn colorless) and then L-Cysteine hydrochloride (act as reducing agent) was added.

A volume of 30 ml of medium was dispensed into 67 ml serum bottles flushed with O_2_-free N_2_. The serum bottles were sealed with butyl rubber stoppers and crimped with aluminum seals. Prior to inoculation, the sealed, pressurized bottles were sterilized in an autoclave at 121°C for 15 min. At time zero, 30 ml of medium was inoculated with 3 ml of the formation fluid/culture under aseptic condition in an anaerobic chamber (Thermo Forma Anaerobic System) operating with mixture of 5%H_2_, 5% CO_2_, 90% N_2_, and background of N_2_. It was also equipped with a palladium catalyst, desiccant, and charcoal to remove excess oxygen. Generally, O_2_ levels equilibrate to 0–5 parts per million (ppm). Inoculated bottles were incubated at respective well’s bottom hole (68–75°C) temperature for 10 days. Bottom hole temperature of the selected oil wells K#35, K#655, J#188, J#31, and J#06 were 68°C, 75°C,70°C, 75°C, and 75°C, respectively.

The produced metabolites such as VFA, biomass and biosurfactant were quantified as described in analytical section. A control set of un-inoculated medium bottles were kept in similar experimental conditions. As consortium J-188 showed highest metabolite production was named as TERIJ-188 in this study. All the experiments were performed in triplicate and the data points are average of the triplicate ± standard deviation (less than 5% of average).

### Analytical Methods

Headspace gas production was quantified by gas chromatography (model GC-7890A, Agilent Ltd., United States) equipped with a molecular sieve packed stainless steel column (2m × 2 mm id NUCON, INDIA) and a thermal conductivity detector (TCD). Argon was used as a carrier gas at a flow rate of 5 ml/min. The operating temperatures of the injection port, oven and the detector were 100, 50, and 150°C, respectively. Prior to the analysis of culture samples, a calibration curve for hydrogen, nitrogen, methane and carbon dioxide was prepared and *R*^2^ value was found to be 0.998 (Rathi et al., 2015). The concentrations of C2–C6 VFAs in liquid phase was analyzed by GC 7890A Agilent Ltd., United States) equipped with flame ionization detector and DB-WAX etr column (30 m × 530 × 1 μm) as described by Rathi et al. (2015). The calibration was performed using mixture of VFA standard (acetic acid, butyric acid, iso butyric acid, valeric acid, iso valeric acid, phosphinic acid, hexanoic acid) and *R*^2^ value was found to be 0.998.

The extraction of biosurfactant was done using solvents, which then precipitated by 1 N HCl and extracted using an equal volume of chloroform: methanol. The organic phase was recovered and dried at 45°C (Vanavil et al., 2013). The presence of carbohydrate in biosurfactant was tested using molish test. For biosurfactant activity, drop collapse assay, oil displacement and emulsification index assay were performed.

The functional group of surfactant was characterized by Fourier Transform Infrared Spectroscopy (Nicolet, 6700) with a scanning wave number from 4000 to 400 cm^-1^. The resolution was set at 4 cm^-1^ with 64 scans per spectrum. The transmission bands were compared with the reported surfactant (Patowary et al., 2017). The extracted biosurfactant was re-dissolved in methanol and the respective ^1^H and ^13^C NMR spectra were recorded at 25°C using a Bruker Avance 300 spectrometer operating at 400.13 MHz. Chemical shifts (d) are given on the ppm scale. The surface tension measurements of culture broth supernatant and biosurfactant solution were performed according to the method described by Kryachko et al. (2016). A CSC DuNouy Tensiometer (Cole Parmer India) equipped with the platinum ring was used. To increase the accuracy of the surface tension measurements, an average of triplicates was determined. All the measurements were performed at room temperature (25°C ± 1°C). The accuracy of surface tension was estimated by using ultra distilled water (72 mN m^-1^) as a control.

### Identification and Characterization of TERIJ-188

To identify TERIJ-188, total genomic DNA was extracted and PCR amplification was done with universal bacterial primers 27F (5′-AGA GTT TGA TCC TGG CTC AG-3′) and 1492R (5′-ACG GCT TAC CTT GTT ACG CTT-3′) as described by Lavania et al. (2015). Clones were processed for cycle sequencing using the BigDye^®^ Terminator v3.1 Cycle Sequencing Kit (Applied Biosystems). The 16S rRNA sequences were checked for purity with Check-Chimera program^1^. Multiple sequence alignments were performed using CLUSTALW, version 1.8 14. A phylogenetic tree was constructed with the evolutionary distances using the neighbor-joining method. Tree topologies were evaluated by performing bootstrap analysis of 1000 data sets with the MEGA version 6.06 packages (Tamura et al., 2013). Sequences of the cultures were submitted to NCBI (National Centre for Biotechnology Information) database. Morphology of TERIJ-188 was studied by SEM (10KV in Zeiss EVO MA 10).

### Effect of Reservoir Conditions on TERIJ-188

The effect of TERIJ-188 was determined in terms of metabolite production at a range of temperature (60, 70, 80, and 90°C), pH (5.0 – 9.0). All experiments were performed for 10 days in 67 ml serum bottles with working volume of 30 ml of liquid anaerobic media. Actively growing culture (cell count 10^5^ cells ml^-1^) was inoculated at 10% (v/v) by using a disposable syringe. The experiment was performed in triplicate and data points are average of the triplicate ± standard deviation (less than 5% of average).

### Core Flooding Test of TERIJ-188

**FIGURE 2:**
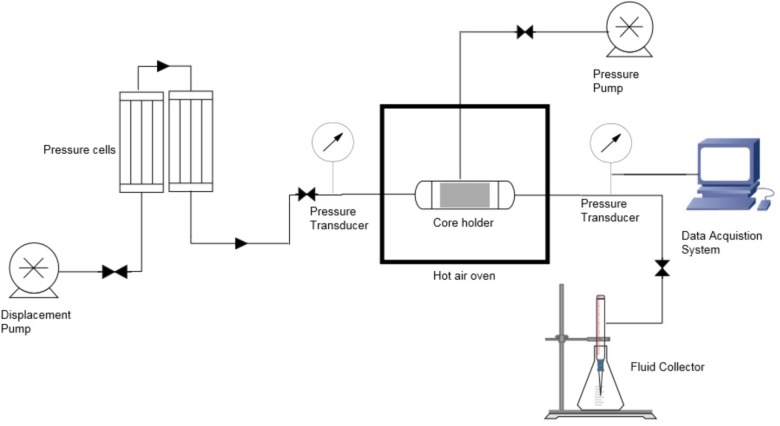
Schematic diagram of core flood apparatus.

The core flood experiment was conducted to determine the water saturation, original oil in place (OOIP), pore volume, porosity, irreducible oil saturation and water permeability at residual oil as shown in **Table 1**. The setup is demonstrated in the **Figure 2**. Edraw software was used to draw the schematic diagram of core flood apparatus which comprise of four components; displacement pump, core holder, pressure cells and fluid collector. The formation water and oil was injected at a constant flow rate through the core which was saturated with oil and water, to mimic the real reservoir condition.

**Table 1:** Petro-physical properties of core flood assay.

Characteristics	Value
**Core flood study**	
Length (cm)	7.80
Diameter (cm)	3.78
Formation water/crude oil	J-188
Pore volume (cc)	19.7 ± 0.04
Porosity (%)	22.5
Water saturation, S_wi_,%PV	27.9 ± 0.02
Original oil in place (OOIP),(ml)	14.2 ± 0.005
Irreducible oil saturation,Sor %PV	33 ± 0.03
Water permeability at residual oil,K_w_@sor (mD)	28.3 ± 0.02
**Operational conditions**	
Temperature	70°C
Pressure,psi	1874
Microbial culture	TERIJ-188
Rate of injection of fluid (cc/min)	0.1

The core was placed in the core holder and saturated with J-188 formation water until saturation. Eventually, the core was saturated with oil. The OOIP was 14.2 ml. Water was flooded by injecting formation water till the point where no oil was coming out. Thereafter, TERIJ-188 consortia along with the optimized media were injected and the set up was incubated at 70°C for 10 days. The reduction in permeability was monitored through the SEM.

The absolute permeability of the column was determined prior to the injection of the bacteria and after the microbial treatment using the falling heat method. The measurement of the ability of the porous media to transmit the fluid is known as absolute permeability, expressed by Darcy’s Law:

*K* = (QμL)/AΔP

where Q is the flow rate, ΔP is a pressure gradient across the core, K is permeability, L is the length of the core holder, A is the cross-sectional area and μ is the viscosity of the fluid. Based on the core flood study, the pressure holding capacity was increased with a decrease in the permeability of the porous material (Borhari et al., 2011).

The percentage of oil recovered was calculated as follows:

Pore Volume (PV) = Volume of brine required to saturate the column
Original Oil in Place (OOIP) = Volume of brine displaced by oil saturation
Initial water saturation % (Swi) = X/PV × 100
Where X = Pore volume –Volume of brine collected after injecting oil
Initial Oil saturation % (Soi) = OOIP/PV × 100
Residual Oil saturation % (Sor) = Xi/OOIP × 100
Where Xi = OOIP-Vol. of oil collected after the water flood
Additional Oil recovery over Sowrf % = (Oil recovered using crude BS)/(Oil in core pack after water flood) × 100

### Pathogenicity Test

The pathogenicity test of TERIJ-188 was studied by acute oral toxicity under EPA 712-C-96-322 OPPTS 885.3550 guidelines. Twelve mice (6 male and 6 female) were allocated to the dose groups: control and inoculum (1 ml) were administrated once by the gauge to mice (Sharma et al., 2017). The mice were fasted overnight and 2 h after administration of the test material. At the end of the inspection period, the surviving experimental animals were sacrificed. Gross necropsy performed and all animals were carefully examined for the presence of anaerobic bacteria. The body weight and organ weight were recorded on test day 0, 7, 14, and 21. All animals were observed for mortality throughout the observation period. RBC (red blood cell), WBC (white blood cell), Hemoglobin, Packed cell Volume, Glucose, BUN (blood urine nitrogen), SGPT (Serum Glutamate Pyruvate Transaminase), Total Proteins and Albumin were studied on 21 days of the study. Kidneys, Spleen, Adrenals, Heart, and Lungs of each animal were weighed after sacrifice. The study was performed by National Toxicology Centre (APT Testing and Research Pvt. Ltd.), Pune.

## Results and Discussion

The leading emphasis of the current study is to implement the BPM in partially depleted oil reservoirs as most of the reservoir have entered into high water cut after initial recovery processes. Improvement in the recovery process is indeed an urgent need. Due to long-time exploitation chemicals, fracturing and acidizing fluids have been injected into the oil reservoir which has been influencing the composition of formation water and also the types and number of microbes in the production wells. Such oil reservoirs in Kalol sand, which is in Ahmedabad-Mehsana tectonic block of North Cambay basin, was considered in the present study. It was discovered in June, 1961 and put on production in mid-sixties. The development of the kalol field is based on various studies over the past 53 years. Formations from such wells were selected because of their high permeability even after long time exploitation.

### Properties of Oil and Formation Fluid

Aiming toward a successful approach for potential residual oil recovery, characteristics of the formation water/oil of the selected oil reservoir was investigated as tabulated in **Table 2**. The formation water was found to be slightly alkaline. The concentration fluoride, sulfate, chloride and heavy metals were in a limit that would facilitate microbial growth. Environmental factors together with the composition of formation water in terms of CHNS plays a significant role in the metabolite production of the selected consortium. TPH of the oil was studied gravimetrically with following composition: 56% aliphatic, 40% aromatic, and 4% NSO and other compounds.

**Table 2:** Physico-chemical analysis of formation water collected from Gujarat oil fields.

Parameter	Method	K#35	K#655	J#06	J#188	J#31
**pH**		7.7 ± 0.02	7.5 ± 0.01	7.6 ± 0.04	7.6 ± 0.02	8.1 ± 0.04
**Anions (mg l^-1^)**						
Chloride	IS3025 Pt 32:1988	560 ± 2.6	300 ± 4.0	750 ± 2.3	1001 ± 3.3	651 ± 1.6
Fluoride	APHA 4500	0.12 ± 0.01	0.55 ± 0.01	0.11 ± 0.01	0.70 ± 0.02	ND
Sulfate	IS3025 Pt 32:1988	44 ± 0.4	119 ± 1.2	43.8 ± 0.5	32.3 ± 0.9	50 ± 0.8
**Elements (ppm)**						
Carbon	APHA:1350	500 ± 1.6	510 ± 4.0	1150 ± 2.6	300 ± 3.7	210 ± 1.2
Hydrogen	APHA:1350	22 ± 0.4	20 ± 0.8	0.11 ± 0.001	140 ± 2.1	80 ± 1.2
Nitrogen	APHA:1350	12 ± 0.5	31 ± 0.8	43.8 ± 0.4	37 ± 0.8	36 ± 0.4
Sulfur	APHA:1350	17 ± 0.2	11 ± 0.2	11.3 ± 0.3	11 ± 0.3	51 ± 1.6
**Heavy metals (mg l^-1^)**						
Arsenic	IS3025 PT 37:1988	ND	ND	ND	ND	ND
Cadmium	APHA3100(B)	ND	ND	ND	ND	ND
Chromium	APHA3500(B)	ND	ND	ND	ND	ND
Copper	APHA3111(B)	ND	ND	ND	ND	ND
Zinc	APHA3100(B)	ND	ND	ND	ND	ND
Silver	APHA3113(B)	ND	ND	ND	ND	ND
Nickel	APHA3111(B)	ND	ND	ND	ND	ND
Iron	APHA3100(B)	0.22 ± 0.001	2.18 ± 0.07	0.12 ± 0.001	0.13 ± 0.002	0.46 ± 0.01

*ND means not detected; standard deviation (±)*.

### Enrichment of Indigenous Anaerobic Consortia

The current study focused on residual oil recovery by targeting high permeability zones of the reservoir. The technique involves three strategies which include injection of microbes capable of producing metabolites, stimulation of thermophilic microbes by injecting selected nutrients and injection of produced *ex situ* biosurfactant directly into the reservoir. Biosurfactants are amphiphilic molecules which reduce interfacial tension between the aqueous phase and hydrocarbons, to mobilize the trapped oil. They also show structural and functional diversity (Banat, 1995; Pornsunthorntawee et al., 2008).

TERIJ-188 is an Indigenous consortium isolated from oil reservoir that contains a blend of different beneficial microorganisms (thermophilic, anaerobic); it is a territory of special bacteria that are living together in synchronization with the rest of nature. The expression “indigenous microorganisms” alludes to a group of microbes that are native to the region, thus named as indigenous (locally existing, or not imported). Further compatibility studies of TERIJ-188, has been conducted and found it suitable with the native population of oil reservoir within the range of bottom hole temperature.

**FIGURE 3:**
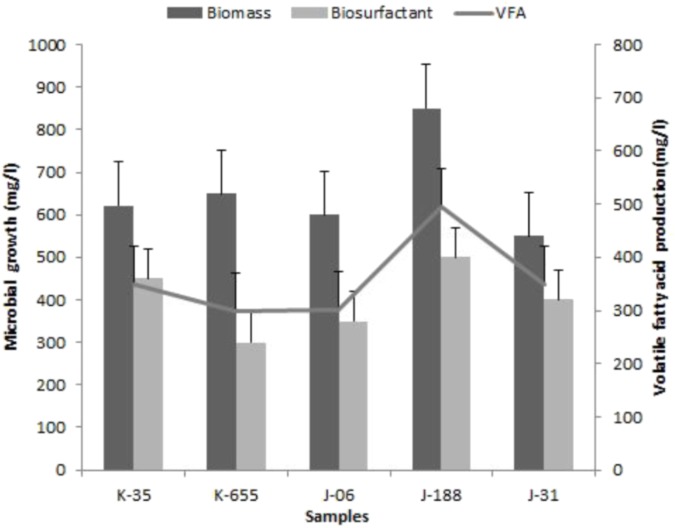
Biomass growth, VFA and bio-surfactant production by isolated consortia K-35, K-655, J-06, J-188, and J-31.

Development of indigenous consortia from the oil reservoir was carried out by enrichment process. After successive enrichments; consortium TERIJ-188 showed significantly higher production of biomass, bio-surfactant and VFA in comparison with the other consortia. Consortium TERIJ-188 showed maximum production of biomass (850 mg l^-1^) as dry cell weight at 70°C in 10 days. Extracted total biosurfactant produced by TERIJ-188 was 500 mg l^-1^ at 70°C in 10 days. Biosurfactant were concentrated for further functional group characterization by FTIR spectroscopy. FT-IR spectra confirmed the presence of aliphatic chain, carboxylic acid, aldehyde, ketones and peptide groups in surfactant produced by TERIJ-188. The major benefit of VFAs is that acid reduces the pH of the environment and can alter fluids – solid surfaces, which ultimately improve the mobility of oil toward the well bore. In the present study, fermentation broth of TERIJ-188 was estimated for VFA production and confirms the presence of acetic acid (495 mg l^-1^) by Gas chromatography at 70°C in 10 days **Figure 3**. Moreover, it validates the ability of the consortium TERIJ-188 toward microbial enhanced oil recovery. Experimental control (un-inoculated media bottles) did not show any VFA production.

In MEOR the microbial biomass involves selective plugging of high permeability zones by the cells that grows within the pore restricting the undesirable water flow through the pores. This forces the water to divert from its path to the smaller pores and relocate the un-swept oil, thus facilitates enhanced oil recovery (Sen, 2008). The residual oil recovery by acid production is one of the mechanisms which play an important role, especially in carbonate reservoirs (Adkins et al., 1992; Adelzadeh et al., 2010).

### Identification and Characterization of TERIJ-188

**FIGURE 4:**
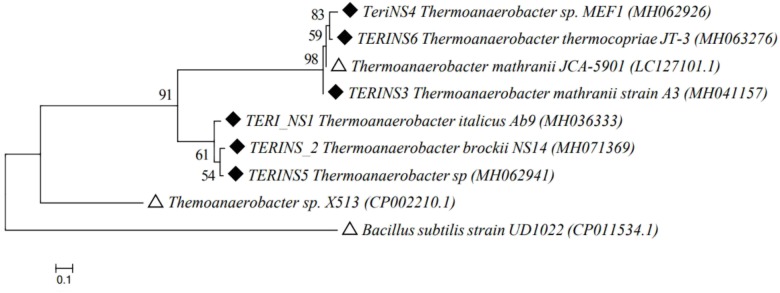
Phylogenetic tree of 16S rRNA gene sequences retrieved from *Thermoanaerobacter* culture TERIJ-188 and closely related species from GenBank database. The topology of tree was obtained by a Neighbor joining method, with bootstrap values (*n* = 1000 replicates) of nodes reported, using MEGA 6.06 software. Scale bar = nucleotide substitutions per site. Outgroup and reference species were represented with Δ whereas the isolated strains were represented with ◆.

Based on the screening data, consortia TERIJ-188 showed significant results. This indicates the potential of TERIJ-188 for enhanced oil recovery, thus the identification and characterization was subsequently taken ahead by 16S rRNA sequencing. The consortia TERIJ-188 showed the presence of five types of bacteria which was *Thermoanaerobacter* sp, *Thermoanaerobacter brockii, Thermoanaerobacter italicus, Thermoanaerobacter mathranii*, and *Thermoanaerobacter thermocopriae.* The nucleotide sequences were submitted to NCBI GenBank with an accession number MH062941, MH071369, MH036333, MH041157, MH063276, and MH062926. All the sequences were aligned using CLUSTALW algorithm. A phylogenetic tree was mapped with the closely related matched based on a Neighbor-Joining method using the Molecular Evolutionary Genetics Analysis (MEGA 6.06) program which showed that the consortium might represent an evident lineage from the known strains of *Thermoanaerobacter* genus in **Figure 4**.

**FIGURE 5:**
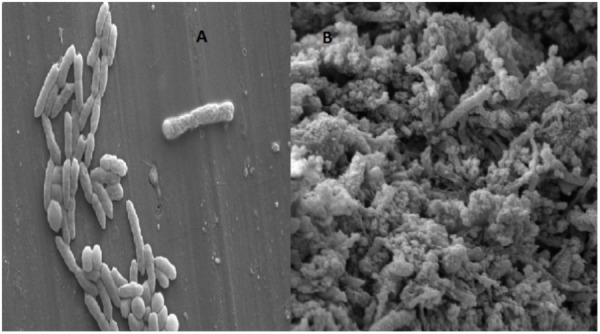
Morphology of the indigenous consortia TERIJ-188. **(A)** Scanning electron micrograph of 5 days grown culture in Baltech media at 15KX magnification, bar 2 μm. **(B)** 10 days grown culture clearly showing criss cross network of bio-surfactant.

Varieties of thermophilic fermentative microorganisms have been isolated from petroleum reservoir ecosystems. Thermophilic fermentative microorganisms mainly belonging to oil fields are either members of the phylum *Thermotoga*e or *Firmicutes. Thermotogae* isolates have been reported as indigenous to petroleum oil reservoir ecosystems by many researchers (Nazina et al., 2006; Zhou et al., 2013). Sogaard et al., 2012 reported the potential of *T. brockii* subsp. and also described its ability for producing biogas, bioacids, biosurfactant and VFA which might play key role in the oil recovery process. Fardeau et al. (2004) reported numerous novel thermophilic species (*Caldanaerobacter subterraneus*) isolated from oil reservoirs. Morphological characterization of enriched consortia TERIJ-188 was performed using Scanning Electron Microscopy. The micrographs of 5 days grown the culture of TERIJ-188 showed rods and cocci (2 μm) and micrograph of 10 days grown consortia clearly showed a criss-cross network of bio-surfactant completely masking the bacterial strains in **Figures 5A,B**.

### Effect of Reservoir Conditions on TERIJ-188

**FIGURE 6:**
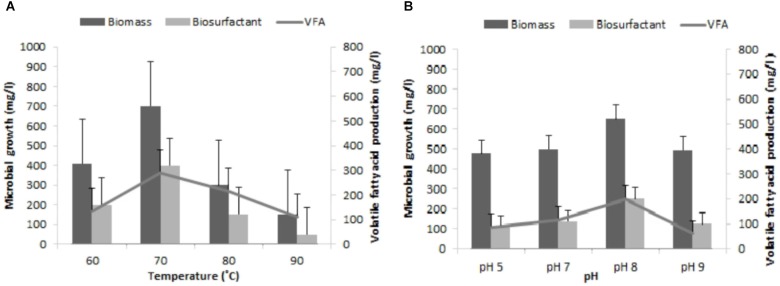
Influence of reservoir conditions on growth of the microbial cell, bio-surfactant and VFA by TERIJ-188 in Baltech medium prepared anaerobically using nitrogen gas. Data was recorded after 10 days of incubation. **(A)** At different temperatures (60, 70, 80, and 90°C). **(B)** At range of pH values (5.0, 7.0, and 9.0).

Various reports show that environmental factors such as temperature, pH, and available nutrients in the reservoir can affect the overall growth of indigenous bacterial community and its metabolite production (Bryant and Lockhart, 2002; Mohan et al., 2010). Hence, for an effective implementation of MEOR in depleted oil reservoirs an attempt was made to optimize the physiological factors (pH, 5.0–9.0 and Temperature, 60–90°C), which plays an important role in the growth and its metabolite generation in the modified M_2_X medium. The indigenous consortium TERIJ-188 developed in this study was isolated from the Jotana oil fields and thus compatible with harsh (extreme) environmental condition of oil fields. The optimum temperature and pH were 70°C and 8.0 (**Figures 6A,B**) for the growth and metabolite generation of TERIJ-188. There are a number of reports on the survival of a microorganism at higher pressure (Rathi et al., 2015).

### Quantitative Estimation of Bio-Surfactant

The efficiency of bio-surfactant produced by TERIJ-188 was estimated by measuring its effect on the surface tension of the media. The surface tension of TERIJ-188 was reduced from 68 to 32 mN m^-1^ after 10 days of incubation at 70°C thus showing stable biosurfactant. The pressure of the reservoir is not limiting factor for the growth of consortia. There are a number of reports on the survival of a microorganism at higher pressure (Rathi et al., 2015). Biosurfactants reduce the surface tension between oil-water/oil-rock interfaces and also alter the wettability of the reservoir rocks which leads to the mobility of oil toward the well bore. The biosurfactant produced in our study further supports the incremental oil recovery (**Supplementary Figures S1, S2**).

### Characterization of Bio-Surfactant by FTIR Spectroscopy

**FIGURE 7:**
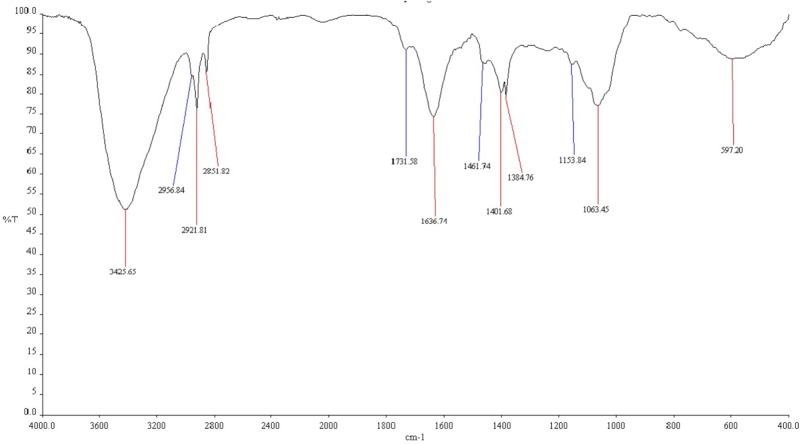
Fourier Transform Infrared spectra of bio-surfactant isolated from TERIJ-188.

The functional groups of bio-surfactant produced by TERIJ-188 were examined by FTIR spectroscopy. An infrared spectrum indicating intense band belonging to N-H stretch was identified at a wave number 3425 cm^-1^ which corresponds to the strong hydrogen bonding. The broad peaks between 3000 and 2900 cm^-1^ were characteristic of the aliphatic chain (–CH_3_, –CH_2_) stretching vibrations. The intense band at 1636 cm^-1^ defined as a linkage between amide I and II indicating the presence of peptide group. The peak at 1401 cm^-1^ was due to the presence of C-N bond. The peak at 1061 cm^-1^ was assigned to O-C-O extend vibrations of carbohydrate confirming the presence of carboxylic acid, aldehyde and ketones (hydroxyl group of hydroxylsate). The vibration at 597 cm^-1^ indicates methylene scissoring vibrations from the proteins in the bacterial filtrate depicted in **Figure 7** (Elazzazy et al., 2015).

### ^1^H and ^13^C NMR Spectroscopy

**FIGURE 8:**
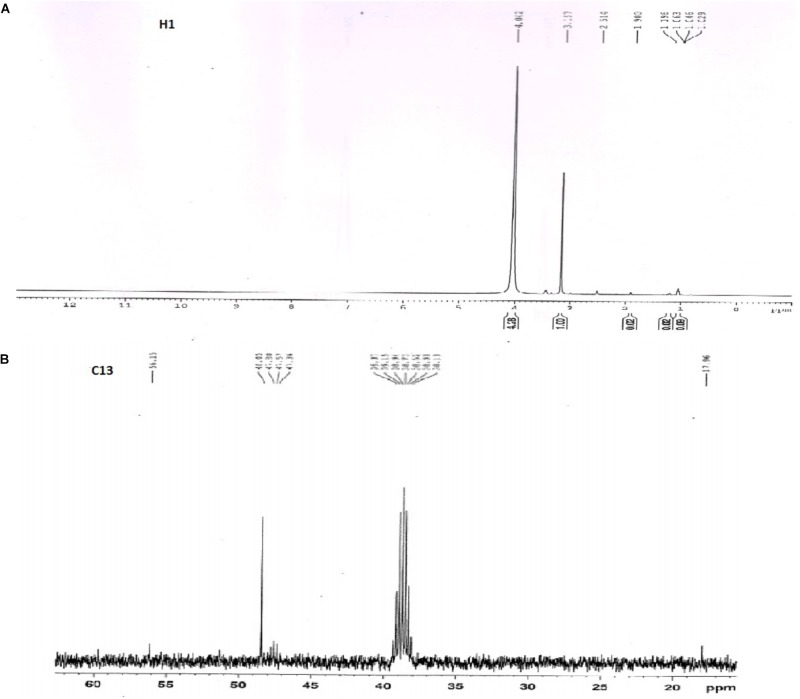
^1^H and ^13^C NMR spectra for extracted biosurfactant. **(A)** ^1^H spectra showed glyceride, ester group and acyl moieties, **(B)** ^13^C spectra represents primary alkyl groups.

The ^1^H and ^13^C NMR results obtained for the extracted biosurfactant was shown in **Figures 8A,B**. The proton resonance is assigned as per the literature. The chemical shift at 4.042 ppm was a methylene group (C1 and C3) of glyceride in triacylglycerols. The moderate intensity band at delta 3.167 ppm represents methyl proton of the ester group. The H-2 protons of acyl moieties at 2.5 ppm and the protons of methylene envelope appear at 1.19 ppm. The ^13^C NMR spectrum shows high-intensity signals at 38.7 and 47.8 ppm and was of primary (RCH2—), secondary (R2CH—) and tertiary (R3C—) alkyl group (Laetitia et al., 2006).

### Core Flood Test

The injection rate was kept constant and injection pressure was monitored regularly. TERIJ-188 was able to alter the permeability of the core sample. The reduction in the permeability is directly proportional to the capability of microbial plugging and its concentration. In MEOR experiment, the optimized nutrient along with microbial consortia TERIJ-188 at exponential phase was injected into the core and incubated at 70°C. After the end of the incubation period, the core was again flooded with oil and water.

The results for the same are summarized below:

Pore Volume (PV) = 19.7 ± 0.04
Sorwf = 5.7 ml ± 0.04
Sorwb = 1.1 ml ± 0.01
Initial water saturation (Swi) = 27.9% ± 0.02 of PV
Swi (%) = 100-72.08 = 27.9% ± 0.02 of PV
Initial oil saturation (Sor) = 33% ± 0.03 of PV
Additional oil recovery over Sorwf (AOR) % = 1.1/5.7 × 100 = 19.2% ± 0.02

**FIGURE 9:**
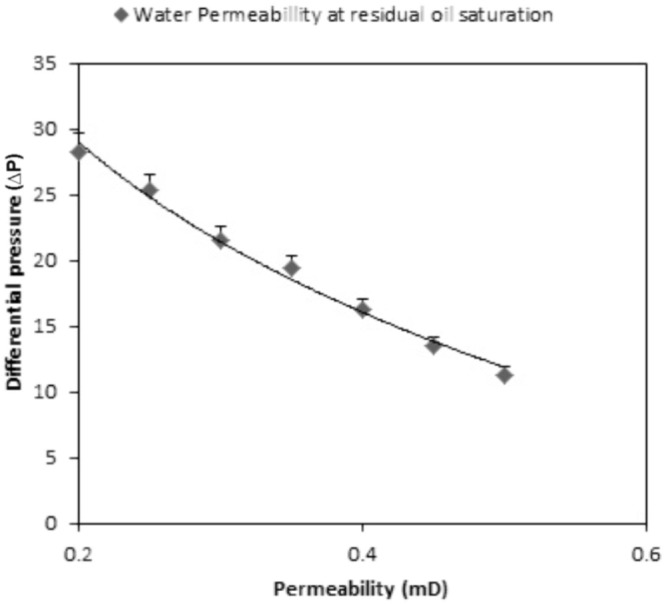
Graphic representation between differential pressure and permeability across the core.

The additional oil recovery was 19.2% of PV. The endpoint permeability was also calculated. Water permeability at the residual oil saturation was reduced to half of its initial point (28.3 to 11.3 mD) and 19.2% incremental oil recovery in a core flood assay. This indicates that TERIJ-188 was clogging the pore space and caused the diversion of water flow path. Selective plugging of the pore space resulted in a gradual increase in the differential pressure across the core as indicated in **Figure 9**. This process improves sweep efficiency (Donio et al., 2013). The results indicated that the inoculated microbial culture TERIJ-188 was effective in releasing oil.

**FIGURE 10:**
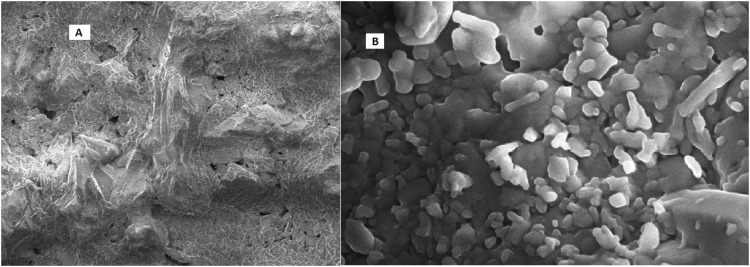
**(A)** SEM image of the untreated core which clearly displays the presence of pores. **(B)** Treated core was indicating the plugging of pores by microbial consortia TERIJ-188 in core flood assay.

After the core flood, the sample of untreated core was further visualized by scanning electron microscopy, which indicates the clear pores in **Figure 10A**. SEM analysis of treated core showed that TERIJ-188 proliferated and produced bio-surfactant in the core of the core flood apparatus. The consortia were clearly visible in the porous media core as depicted in **Figure 10B**. The bio-surfactant acts as an emulsifier and facilitates the biomass to adhere to the solid surface (Kowalewski et al., 2006). The previous reports show that bio-surfactant producing microbes were isolated from reservoir formation water. These microbes have a tendency to enhance the oil recovery by reducing interfacial surface tension (Nerurkar et al., 2009).

Incremental oil recovery via plugging potential of TERIJ-188 was demonstrated in core flood experiment. There is an inverse correlation between permeability and pressure as the trend can be seen in the results where permeability was decreased and the pressure was increased. Reduction in permeability was clearly evident for the plugging of pores. A core flood experiment was performed using *Enterobacter cloacae* and *Enterobacter hormaechei* which led to 6.4% increment in oil recovery under mesophilic conditions (Gudina et al., 2013; Rabiei et al., 2013). Nerurkar et al. (2009) isolated *Bacillus licheniformis* TT33 from hot water spring. Its biosurfactant reduces the surface tension from 72 to 34 mN m^-1^. In India, joint research of ONGC, IRS, and TERI leads to the improved Microbial technology of cultured set of microbes that could survive temperatures as high as 90°C. These microbes were effectively tested in oil wells of Gujarat and Assam. The increment of threefold in oil recovery has been reported^2^.

Our previous studies Rathi et al. (2015) also showed a significant enhancement in oil recovery at 70°C. Castorena-Cortés et al. (2012) also established a mixed culture of *Thermoanaerobacter* sp. having the ability to produce gasses, VFA (693 mg l^-1^) and solvent (as ethanol 173 mg l^-1^) showing 12% of heavy oil recovery in 10 days (Castorena-Cortés et al., 2012).

Similarly, the metabolites produced by TERIJ-188 lead to 19.2% residual oil recovery at 70°C along with the permeability reduction from 28.3 to 11.3 mD. Therefore, Consortium TERIJ-188 has massive potential in residual oil recovery application. It produces biomass, which clog the permeable zones, gasses led to re-pressurization of oil reservoirs as well as organic acids. Thus, enhances oil mobility from wells in lesser time.

### Pathogenicity Assessment

TERIJ-188 consortia did not show any case of mortality in the orally treated mice (1 ml of dose containing 1 × 10^6^ CFU). All the experimental mice were normal until the end of this investigation. The gain in the body weight of treated mice was not adversely affected during 21 days of inception. There was no statistically significant difference in the hematological parameters – Red Blood Cells, White Blood Cells, Hemoglobin, Packed Cell Volume, and blood chemistry parameters – glucose, Blood Urea Nitrogen, total proteins and albumin in the test group. Except for all the reported results, there was no statistical effect observed in SGPT, its mean concentration was 46.8 International Unit/Liter in tested animal and control showed around 45.3 International Unit/Liter. The results from the necropsy revealed no abnormalities were observed or detected in the test when compared with the control group animals. The consortia did not induce any gross pathological alterations in experimental models during their necropsy. The sacrificed mice’s organs were thoroughly examined and were completely free from any live anaerobic bacteria. The consortia were non-toxic and non-virulent. Hence, consortium (TERIJ-188) found safe for field implementation (**Supplementary Tables S1–S3**).

## Conclusion

The isolated consortia (TERIJ-188) have the potential toward enhance oil recovery in the high temperature reservoir by BPM. The enrichment process led to the growth of anaerobic, halo and thermo tolerant microbes. TERIJ-188 was elected due to its capability to produce the substantial amount of biomass and secondary metabolites (VFA, biosurfactant). Furthermore, TERIJ-188 had an added advantage of reducing the surface tension (68 to 32 mN m^-1^). The microorganism in the TERIJ-188 was identified to be *Thermoanaerobacter* sp. The produced bio-surfactant was characterized by FTIR and NMR that revealed the presence of aliphatic chain, triacylglycerols. The potential of TERIJ-188 was tested in core flood to mobilize oil from the low permeable zones. In the core flood assay, the produced biomass interacted with the oil entrapped in the porous medium and overall oil mobility 19.2%. This study validates the importance of TERIJ-188 for industrial application.

## Ethics Statement

The pathogenicity was studied by acute oral toxicity under EPA 712-C-96-322 OPPTS 885.3550 guidelines. The study was performed at National Toxicology Centre (APT Testing and Research Pvt. Ltd.), Pune. The report of toxicity test conducted has been submitted through study code No. MS0111/1617/0862a. APT Testing and Research Pvt. Ltd., is approved by Food & Drug Administration, Maharashtra State, Pune Through License No. 37-PD/TL/7.

## Author Contributions

NS and ML conceived and designed the experiments. VK, ML, and BL provided all the resources for performing experiments. NS performed the experiments. VK analyzed the data and core flood experiments were conducted. BL and ML critically reviewed the manuscript. NS and ML for important intellectual content. All authors approved the final version to be published.

## Conflict of Interest Statement

The authors declare that the research was conducted in the absence of any commercial or financial relationships that could be construed as a potential conflict of interest.

## Footnotes

1 http://rdp.cme.msu.edu/

2 http://www.otbl.co.in/Case-Studies-meor.php
