# Supplementary material for: Laboratory Investigation of Indigenous Consortia TERIJ-188 for Incremental Oil Recovery
Source: Front Microbiol. 2018 Oct 9;9:2357. doi: 10.3389/fmicb.2018.02357 (PMC6189299; doi:10.3389/fmicb.2018.02357)
Supplement: Supplementary file 1 [file Data_Sheet_1.docx]

**Supplementary material**

**Experiment:** **Evaluation of biosurfactant for incremental oil recovery**

**Methodology**

The oil spreading method was used to quantify the amount of biosurfactant present in the sample. A petri dish was filled with 30 ml of deionized water and 30 µl of crude oil was added on the water which forms oil film. 10µl of culture surfactant was poured on the surface of the oil film named as treated. And in the control, 10 µl of deionized water was added on the surface of oil film.

**Result**


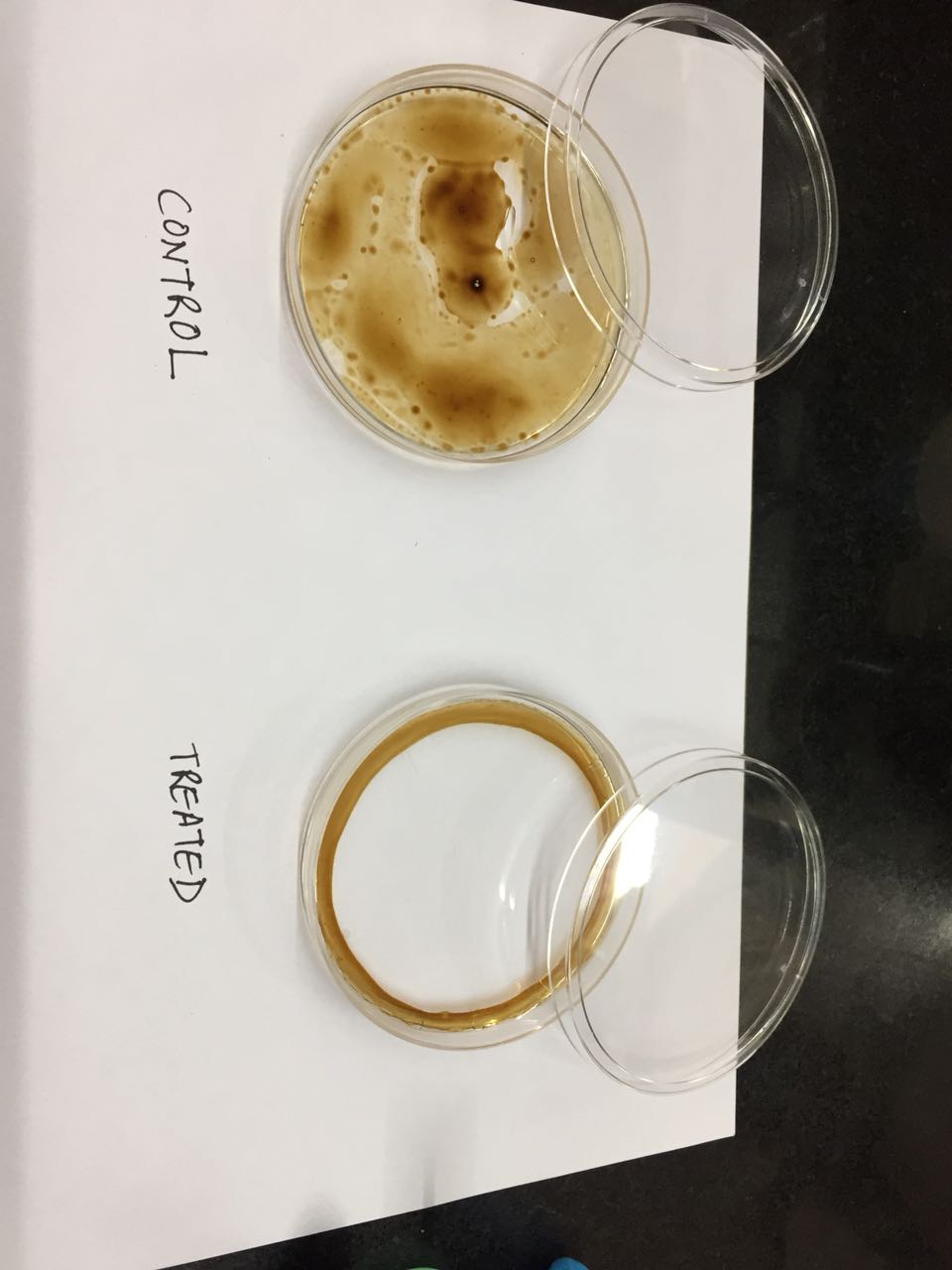
The activity of biosurfactant was recorded as the diameter of clear zone formed on the oil film (Fig. S.1). It was clearly evident that the biosurfcatant produced by TERIJ-188 was effective and can be used in reducing the interfacial tension between the oil-water and oil-rock.

FIGURE S1. Oil spreading test of extracted bio-surfactant

**Experiment:** **Screening of biosurfactant via emulsification index**

**Methodology**

The emulsification index assay was performed. In this assay, 2 ml of olive oil and 2 ml of biosurfactant was added in to the test tubes. The emulsion was vortexed for 3 mins; and after 24 hours, the volume of emulsion was measured by taking the layer formed between the biosurfactant and the oil. Saline solution was served as a negative control. The emulsion index was calculated by using the following equation:

$$\%E24=Height of emulsion formed\div Total height of the solution\times100$$

**Result**

The biosurfactant shows a substantial emulsification capacity, suggesting the potential of surfactant to produce emulsion and improve the mobilization of oil through porous areas. The biosurfactant displays the stability as EI remain constant during the test time. Biosurfactant have emulsification activity that was clearly shown in Fig. S. 2.


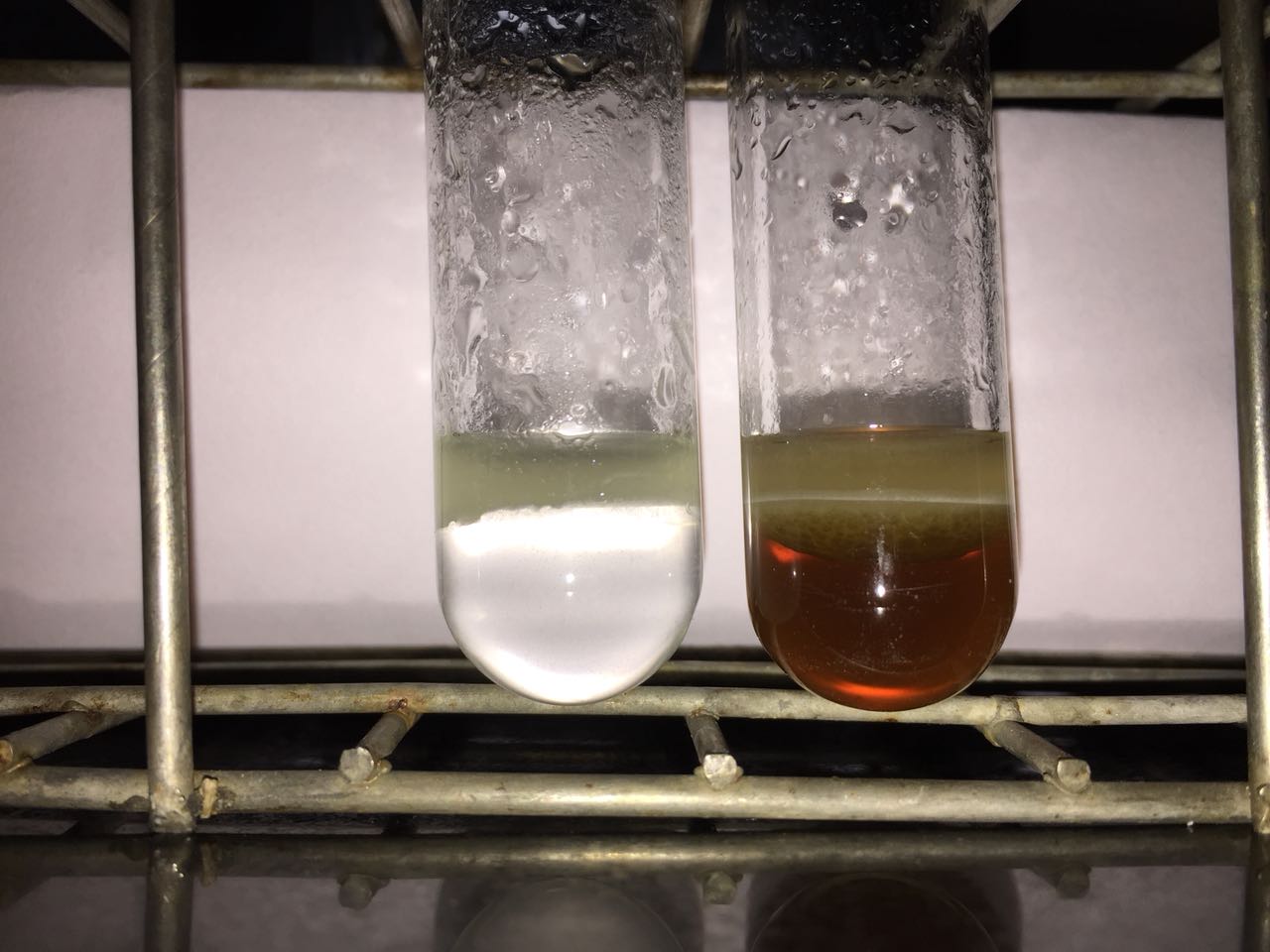


Formation of emulsion

FIGURE S2. Bio-surfactant depicts positive emulsification index; left side tube was serve as negative control (saline was added)

**Experiment:** **Investigation of pathogenicity of isolated consortia**

**Methodology**

The pathogenicity was investigated through acute oral toxicity to access its pathogenicity. The consortia were administered to mice (both female and male) and were observed for 21 days.

**Results**

All the experimental mice were normal till the end of the investigation. The gain in the body weight of treated mice was not adversely affected during 21 days of inception. The results from necropsy revealed no abnormalities were observed or detected in the test when compared with the control group animals (Table S1).

| **Groups** | **Days of observation** | | | | | | | | | | | | | | | | | | | | |
| --- | --- | --- | --- | --- | --- | --- | --- | --- | --- | --- | --- | --- | --- | --- | --- | --- | --- | --- | --- | --- | --- |
|  | **1** | **2** | **3** | **4** | **5** | **6** | **7** | **8** | **9** | **10** | **11** | **12** | **13** | **14** | **15** | **16** | **17** | **18** | **19** | **20** | **21** |
| Control  Test | N  N | N  N | N  N | N  N | N  N | N  N | N  N | N  N | N  N | N  N | N  N | N  N | N  N | N  N | N  N | N  N | N  N | N  N | N  N | N  N | N  N |

**Table S1** Mean clinical mortality data of control and test

N stands for Normal

There was no statistically significant difference in blood chemistry parameters such as glucose, SGPT, total proteins and Blood Urea Nitrogen in the test and control group (Table S2).

**Table S2** Indicating the blood chemistry parameter in control and test mice

| **Group** | **Glucose**  **(mg %)** | **SGPT (IU/l)** | **Total Protein (g %)** | **Blood Urea Nitrogen (mg %)** |
| --- | --- | --- | --- | --- |
| Control | 96.7 | 46.8 | 6.2 | 30 |
| Test | 100 | 45.3 | 6 | 30 |

There was no statistically significant difference in the haematological parameters – Red Blood Cells, White Blood Cells, Hemoglobin and Packed Cell Volume (Table S3). The consortia were appeared to be non-pathogenic and non –virulent.

**Table S3** Indicating hematological parameters in control and test mice

| **Group** | **Red blood cells (million/ cmm)** | **White blood cells(thousand/ cmm)** | **Hemoglobin**  **(g %)** | **Packed cell volume**  **(%)** |
| --- | --- | --- | --- | --- |
| Control | 6.89 | 2.2 | 8.4 | 29.4 |
| Test | 7.89 | 5.6 | 10.1 | 33.2 |

**Reference**

Youssef, N. H., Duncan, K. E., Nagle, D. P., Savage, K.N., Knapp,R.M., and McInerney, M.J., (2004). Comparsion of methods to detect biosurfactant production by diverse micro-organisms. J. Microbiol Methods.56,339-47.

Yela,A.C.A.,Martinez,M.A.T.M.,Pineros,G.A.R.,Lopez,V.C.,Villamizar,S.H.,Velez,V.L.N., and Abraham,W.R., (2016). A comparsion between conventional Pseudomonas aeruginosa rhamnolipids and E. coli transmembrane proteins for oil recovery enhancing. 112, 59-65.
